# Early prediction and identification for severe patients during the pandemic of COVID-19: A severe COVID-19 risk model constructed by multivariate logistic regression analysis

**DOI:** 10.7189/jogh.10.020510

**Published:** 2020-12

**Authors:** Haifeng Hu, Hong Du, Jing Li, Yage Wang, Xiaoqing Wu, Chunfu Wang, Ye Zhang, Gufen Zhang, Yanyan Zhao, Wen Kang, Jianqi Lian

**Affiliations:** Center for Infectious Diseases, Second Affiliated Hospital of Air Force Medical University, Xi’an, Shaanxi, China

## Abstract

**Background:**

As an emergent and fulminant infectious disease, Corona Virus Disease 2019 (COVID-19) has caused a worldwide pandemic. The early identification and timely treatment of severe patients are crucial to reducing the mortality of COVID-19. This study aimed to investigate the clinical characteristics and early predictors for severe COVID-19, and to establish a prediction model for the identification and triage of severe patients.

**Methods:**

All confirmed patients with COVID-19 admitted by the Second Affiliated Hospital of Air Force Medical University were enrolled in this retrospective non-interventional study. The patients were divided into a mild group and a severe group, and the clinical data were compared between the two groups. Univariate and multivariate analysis were used to identify the independent early predictors for severe COVID-19, and the prediction model was constructed by multivariate logistic regression analysis. Receiver operating characteristic (ROC) curve was used to evaluate the predictive value of the prediction model and each early predictor.

**Results:**

A total of 40 patients were enrolled in this study, of whom 19 were mild and 21 were severe. The proportions of patients with venerable age (≥60 years old), comorbidities, and hypertension in severe patients were higher than that of the mild (*P* < 0.05). The duration of fever and respiratory symptoms, and the interval from illness onset to viral clearance were longer in severe patients (*P* < 0.05). Most patients received at least one form of oxygen treatments, while severe patients required more mechanical ventilation (*P* < 0.05). Univariate and multivariate analysis showed that venerable age, hypertension, lymphopenia, hypoalbuminemia and elevated neutrophil lymphocyte ratio (NLR) were the independent high-risk factors for severe COVID-19. ROC curves demonstrated significant predictive value of age, lymphocyte count, albumin and NLR for severe COVID-19. The sensitivity and specificity of the newly constructed prediction model for predicting severe COVID-19 was 90.5% and 84.2%, respectively, and whose positive predictive value, negative predictive value and crude agreement were all over 85%.

**Conclusions:**

The severe COVID-19 risk model might help clinicians quickly identify severe patients at an early stage and timely take optimal therapeutic schedule for them.

Currently, Corona Virus Disease 2019 (COVID-19) which caused by severe acute respiratory syndrome coronavirus 2 (SARS-CoV-2) is rapidly spreading and wreaking havoc all over the world [1]. As of May 24, 2020, more than 5 000 000 COVID-19 patients and asymptomatic SARS-CoV-2 infected individuals had been reported in more than 200 countries and regions [2]. With the accumulation of clinical experience, more and more detailed information about COVID-19 has been revealed. Although previous studies have shown that most COVID-19 patients have a favorable clinical outcome, some severe patients may manifest dyspnea and hypoxemia within 1 week after illness onset, and which may quickly progress to acute respiratory distress syndrome (ARDS) or respiratory failure [3-15]. Additionally, the surging patients have caused a significant shock and challenge to the entire health care system. Consequently, the efficient triage of patients according to the severity of COVID-19 so as to choose appropriate treatment is vital for the rational use of the limited medical resources [6].

Previous studies have shown that older age, comorbidities, hypertension, lymphopenia, and elevated D-dimer, lactate dehydrogenase (LDH), serum ferritin, IL-6, SOFA score, MuLBSTA Score were associated with the disease progression, ARDS present or poor prognosis of COVID-19 patients [4-10,12,14]. However, some of the studies [4,5,7,12] mentioned above were univariate analysis-based studies, and lack of the delineation of virological transformation course and the identification or evaluation of early predictors for severe COVID-19. In this study, we elaborated the details of clinical features and virological transformation course of the enrolled patients, as well as ascertained potential early predictors and further constructed a prediction model for severe COVID-19. We hope the results of this study could help the clinicians implement the triage of patients with COVID-19 more efficiently and choose the best treatment schedule for the patients.

## METHODS

### Study population

In this retrospective non-interventional study, we enrolled all patients with COVID-19 admitted by the Second Affiliated Hospital of Air Force Medical University from January 24, 2020 to March 26, 2020. All enrolled patients were over 18 years old and had a definite clinical outcome (discharged or death), and were confirmed by positive SARS-CoV-2 RNA in nasopharyngeal swab specimens using real-time reverse-transcriptase polymerase chain reaction assay. The confirmative examination was conducted by Xi’an municipal Center for Diseases Prevention and Control according to the same protocol which has been described previously [11].

### Procedures

All patients were diagnosed and clinically typed according to the “Diagnosis and Treatment Protocol for Novel Coronavirus Pneumonia (Trial Version 7)” issued by the National Health Commission of China [16], and were divided into a mild group (patients of mild type and moderate type) and a severe group (patients of serious type and critical type). The classification of all patients was confirmed by their attending physicians respectively. (The specific grouping criteria were showed in Table S1 of the **Online Supplementary Document**)

The clinical data of demographic, epidemiological, symptoms and signs, laboratory, treatments and outcomes were extracted from the electronic medical records by two physicians who had been involved in the treatment of COVID-19 patients, and which were confirmed independently by at least two researchers. In order to protect the privacy of patients, we hid the identity information of all patients in the process of data collection.

Laboratory data in this study mainly include routine blood tests, biochemistry tests, blood clotting tests and infection-related indices. Most of these laboratory tests were conducted on admission, and the frequency of subsequent detections was determined by the attending physician according to the patient's condition. The initial and extreme values of these laboratory indexes were collected for analysis in this study. The initial values of laboratory tests on admission were used to explore early predictors and construct prediction model for severe COVID-19. All of the initial values were obtained before the date when the clinical classification of patients was determined. The nasopharyngeal swab specimens of patients were obtained every other day after hospitalization, which were used to SARS-CoV-2 RNA re-examination by the clinical laboratory department of our hospital, but only qualitative results were obtained.

### Definitions

Exposure history was defined as with a definite history of travelling to Wuhan or exposure to individuals with confirmed or suspected SARS-CoV-2 infection within two weeks before the onset of illness. The incubation period was defined as the interval from exposure to illness onset, which was estimated among the patients who could provide the exact date of intimate contact with confirmed or suspected SARS-CoV-2 infection individuals. Comorbidity was defined as having at least one of the followings: hypertension, diabetes, coronary heart disease, chronic obstructive pulmonary disease (COPD), cerebral infarction, anemia and carcinoma. Since not all laboratory tests could be performed immediately after admission, the initial value was the result of the first test within 48 hours after admission. Extreme value referred to the maximum or minimum value of laboratory tests during hospitalization. The negative results of two consecutive SARS-CoV-2 RNA detections which were taken apart more than 24 hours were considered as viral clearance. Viral clearance, axillary temperature below 37.3°C for more than 3 days, obvious alleviation of respiratory symptoms, and significant improvement of exudative lesions on pulmonary imaging were the discharge criteria of patients, all of which were indispensable.

### Ethics statement

This retrospective non-interventional study was approved and granted a waiver of written informed consent by the ethics committee of the Second Affiliated Hospital of Air Force Medical University, and which was performed in accordance with the Helsinki Declaration.

### Statistical analysis

Given the small sample size of this study, continuous and categorical variables were presented as median (interquartile range) and numbers (percentage), and were compared by Mann-Whitney U test and Fisher exact test, respectively. The demographics and initial laboratory indexes with significant differences between the two groups were assessed by univariate and multivariate logistic regression analysis to explore the independent early predictors and risk factors associated with the disease severity of COVID-19. The Kaplan-Meier survival analysis and COX regression analysis were used to investigate the independent adverse factors which could obstruct the recovery and discharge of patients with COVID-19. The independent risk factors and early predictors for severe COVID-19 were finally ascertained based on the results of the above statistical analyses. The predictive efficacy of each early predictor was measured by receiver operating characteristic (ROC) curves. A two-sided *P* < 0.05 was considered statistically significant. All statistical analyses were performed using SPSS Statistics 23.0 software (IBM Inc, Chicago IL, USA).

## RESULTS

### Demographics, epidemiological and clinical characteristics

A total of 40 patients with a median age of 51.0 (42.0-66.8) years were enrolled in this study, including 16 females and 24 males (**Table 1**). According to the grouping criteria mentioned above, there were 19 mild cases and 21 severe cases. The median age of severe group was older than that of the mild, and also with a higher proportion of over 60 years old (**Table 1**). Nearly half of the patients suffered from comorbidities, with hypertension being the most common comorbidity, and which was more prominent in severe patients (**Table 1**). The median incubation period was 5.5 (3.0-10.0) days, which was estimated based on the information submitted by the patients who could provide accurate exposure date (**Table 1**).

**Table 1:** Clinical features of patients with COVID-19

	All patients (n = 40)	Mild group (n = 19)	Severe group (n = 21)	*P* value
**Demographic and epidemiologic characteristics**				
Gender	-	-	-	0.796
-Female	16 (40.0%)	8 (42.1%)	8 (38.1%)	-
-Male	24 (60.0%)	11 (57.9%)	13 (61.9%)	-
Age, years	51.0 (42.0-66.8)	43.0 (36.0-64.0)	63.0 (48.5-70.0)	0.008
<60	23 (57.5%)	14 (73.7%)	9 (42.9%)	0.049
≥60	17 (42.5%)	5 (26.3%)	12 (57.1%)	
Exposure history	31 (77.5%)	15 (78.9%)	16 (76.2%)	1.000
Smoking history	11 (27.5%)	3 (15.8%)	8 (38.1%)	0.115
Comorbidities	18 (45.0%)	4 (21.1%)	14 (66.7%)	0.004
Hypertension	13 (32.5%)	3 (15.8%)	10 (47.6%)	0.032
Diabetes	6 (15.0%)	2 (10.5%)	4 (19.0%)	0.664
Coronary heart disease	2 (5.0%)	1 (5.3%)	1 (4.8%)	1.000
COPD	2 (5.0%)	0 (0.0%)	2 (9.5%)	0.488
Anemia	1 (2.5%)	0 (0.0%)	1 (4.8%)	1.000
Cerebral infarction	3 (7.5%)	0 (0.0%)	3 (14.3%)	0.233
Carcinoma	1 (2.5%)	0 (0.0%)	1 (4.8%)	1.000
Length of incubation, days	5.5 (3.0-10.0)	6.0 (3.0-10.0)	5.0 (2.5-10.0)	0.653
Time from illness onset to hospital admission, days	4.5 (2.0-7.0)	4.0 (2.0-7.0)	5.0 (1.5-8.0)	0.397
Length of hospitalization, days	26.5 (16.5 − 37.5)	17.0 (12.0 − 27.0)	37.0 (26.0 − 42.0)	<0.001
Time from illness onset to discharge or death, days	30.0 (23.5 − 43.5)	24.0 (17.0 − 28.0)	41.0 (30.0 − 49.5)	<0.001
Time from illness onset to the first time SARS-CoV-2 RNA positive, days	4.0 (1.5 − 6.5)	3.0 (2.0 − 8.0)	4.0 (1.0-6.5)	0.794
Time from illness onset to viral clearance, days	15.5 (9.5-26.5)	12.0 (7.0-17.0)	21.0 (13.0-41.0)	0.005
**Symptoms and signs**				
Fever	37 (92.5%)	16 (84.2%)	21 (100.0%)	0.098
Fatigue	28 (70.0%)	10 (52.6%)	18 (85.7%)	0.023
Cough	26 (65.0%)	9 (47.4%)	17 (81.0%)	0.026
Pharyngalgia	2 (5.0%)	2 (10.5%)	0 (0.0%)	0.219
Expectoration	4 (10.0%)	0 (0.0%)	4 (19.0%)	0.108
Polypnea	21 (52.5%)	5 (26.3%)	16 (76.2%)	0.002
Diarrhoea	3 (7.5%)	0 (0.0%)	3 (14.3%)	0.233
Nausea or vomiting	7 (17.5%)	3 (15.8%)	4 (19.0%)	1.000
Duration of fever, days	7.5 (3.0-12.0)	4.0 (1.0-9.0)	10.0 (6.0-13.5)	0.008
Duration of respiratory symptoms, days	11.5 (1.0-33.0)	5.0 (0.5-12.0)	26.0 (9.0-46.5)	0.001
Highest axillary temperature, °C	38.2 (37.8-38.9)	37.9 (37.5-38.6)	38.5 (38.0-39.1)	0.052
Pulse >120 beats per min	9 (22.5%)	0 (0.0%)	9 (42.9%)	0.001
Respiratory rate >24 breaths per min	19 (47.5%)	2 (10.5%)	17 (81.0%)	<0.001
Systolic blood pressure <90 mm Hg	3 (7.5%)	0 (0.0%)	3 (14.3%)	0.233
Oxygen saturation <94%	19 (47.5%)	1 (5.3%)	18 (85.7%)	<0.001
Imaging features*				
Imaging anomaly	38 (95.0%)	17 (89.5%)	21 (100.0%)	0.219
Extent of imaging infiltration	-	-	-	0.086
No imaging infiltration	2 (5.0%)	2 (10.5%)	0 (0.0%)	-
Unilateral	2 (5.0%)	2 (10.5%)	0 (0.0%)	-
Bilateral	36 (90.0%)	15 (78.9%)	21 (100.0%)	-
**Laboratory findings†**				
White blood cell count, ×10^9^/L	5.54 (3.63-8.16)	4.96 (3.79-6.28)	5.81 (3.40-9.90)	0.171
<4	12 (30.0%)	6 (31.6%)	6 (28.6%)	0.070
4-10	23 (57.5%)	13 (68.4%)	10 (47.6%)	
>10	5 (12.5%)	0 (0.0%)	5 (23.8%)	
Lymphocyte count, ×10^9^/L	0.76 (0.45-1.34)	1.27 (0.74-1.90)	0.47 (0.37-0.82)	<0.001
<0.8	21 (52.5%)	5 (26.3%)	16 (76.2%)	0.002
Neutrophil count, ×10^9^/L	3.90 (2.68-5.63)	3.06 (2.37-4.34)	5.13 (2.87-9.12)	0.024
<2.5	10 (25.0%)	5 (26.3%)	5 (23.8%)	0.144
2.5-7.5	23 (57.5%)	13 (68.4%)	10 (47.6%)	
>7.5	7 (17.5%)	1 (5.3%)	6 (28.6%)	
Neutrophil lymphocyte ratio	4.54 (2.61-11.09)	2.94 (1.70-4.94)	8.64 (4.21-20.85)	<0.001
Platelet count, ×10^9^/L	213.00 (171.50 − 261.25)	210.00 (173.00 − 272.00)	216.00 (170.00 − 245.00)	0.441
<100	2 (5.0%)	0 (0.0%)	2 (9.5%)	0.488
Haemoglobin, g/L	135.00 (127.75 − 140.75)	138.00 (133.00 − 141.00)	133.00 (116.00 − 139.00)	0.324
<120	3 (7.5%)	0 (0.0%)	3 (14.3%)	0.233
Albumin, g/L	38.45 (34.83 − 42.53)	42.00 (38.70-44.10)	35.80 (32.35-38.65)	<0.001
<35	11 (27.5%)	1 (5.3%)	10 (47.6%)	0.003
Globulin, g/L	33.15 (30.03-36.15)	31.90 (29.20-34.90)	33.80 (30.20-37.80)	0.119
>32	22 (55.0%)	8 (42.1%)	14 (66.7%)	0.119
Alanine aminotransferase, U/L	38.00 (29.25-62.00)	32.00 (28.00-39.00)	57.00 (35.50-69.50)	0.018
>40	18 (45.0%)	4 (21.1%)	14 (66.7%)	0.004
Aspartate aminotransferase, U/L	35.50 (29.00-54.75)	33.00 (24.00-40.00)	50.00 (33.50-70.50)	0.013
>40	17 (42.5%)	4 (21.1%)	13 (61.9%)	0.009
Total bilirubin, μmol/L	15.35 (12.04-19.05)	13.60 (11.95-17.10)	17.10 (11.99-24.10)	0.176
>17.1	13 (32.5%)	3 (15.8%)	10 (47.6%)	0.032
Blood urea nitrogen, mmol/L	5.47 (3.95-6.15)	4.80 (3.45-5.20)	6.00 (4.85-7.30)	0.094
>7.5	4 (10.0%)	0 (0.0%)	4 (19.0%)	0.108
Creatinine, μmol/L	61.00 (48.98-84.85)	58.20 (48.40-77.50)	75.50 (50.35-88.35)	0.379
>104	1 (2.5%)	1 (5.3%)	0 (0.0%)	0.475
C-reactive protein, mg/L	12.14 (4.20-32.20)	7.14 (3.20-24.51)	21.12 (8.40-55.71)	0.031
>5	28 (70.0%)	11 (57.9%)	17 (81.0%)	0.112
**Treatments**				
High-flow nasal cannula oxygen therapy	25 (62.5%)	8 (42.1%)	17 (81.0%)	0.011
Mechanical ventilation	13 (32.5%)	0 (0.0%)	13 (61.9%)	<0.001
Continuous renal replacement therapy	2 (5.0%)	0 (0.0%)	2 (9.5%)	0.488
Extracorporeal membrane oxygenation	2 (5.0%)	0 (0.0%)	2 (9.5%)	0.488
Antiviral treatment‡	39 (97.5%)	19 (100.0%)	20 (95.2%)	1.000
Duration of antiviral treatment, days	7.5 (4.0-10.0)	4.0 (3.0-10.0)	9.0 (7.0-10.0)	0.047
Antibiotics	38 (95.0%)	17 (89.5%)	21 (100.0%)	0.219
Corticosteroids	33 (82.5%)	14 (73.7%)	19 (90.5%)	0.226
Immunomodulator§	30 (75.0%)	11 (57.9%)	19 (90.5%)	0.028
Intravenous albumin infusion	8 (20.0%)	0 (0.0%)	8 (38.1%)	0.004
Interferon alpha inhalation	35 (87.5%)	14 (73.7%)	21 (100.0%)	0.018
Traditional Chinese medicine	25 (62.5%)	11 (57.9%)	14 (66.7%)	0.567
**Clinical outcomes**				
Complications	15 (37.5%)	0 (0.0%)	15 (71.4%)	<0.001
ARDS	9 (22.5%)	0 (0.0%)	9 (42.9%)	0.001
Respiratory failure	13 (32.5%)	0 (0.0%)	13 (61.9%)	<0.001
Secondary infection	8 (20.0%)	0 (0.0%)	8 (38.1%)	0.004
Septic Shock	2 (5.0%)	0 (0.0%)	2 (9.5%)	0.488
Acute cardiac injury or heart failure	4 (10.0%)	0 (0.0%)	4 (19.0%)	0.108
Coagulopathy	4 (10.0%)	0 (0.0%)	4 (19.0%)	0.108
Acute kidney injury	3 (7.5%)	0 (0.0%)	3 (14.3%)	0.233
MODS	2 (5.0%)	0 (0.0%)	2 (9.5%)	0.488
ICU admission	9 (22.5%)	0 (0.0%)	9 (42.9%)	0.001
Death	2 (5%)	0 (0.0%)	2 (9.5%)	0.488

COPD - chronic obstructive pulmonary disease, ARDS - acute respiratory distress syndrome, MODS - multiple organ dysfunction syndrome

*Imaging features: including chest x-ray and computed tomography scan.

†All laboratory indexes were the initial values on admission.

‡Antiviral treatment: including Lopinavir/Ritonavir or Arbidol.

§Immunomodulator: including thymosin and gamma globulin.

**Figure 1:**
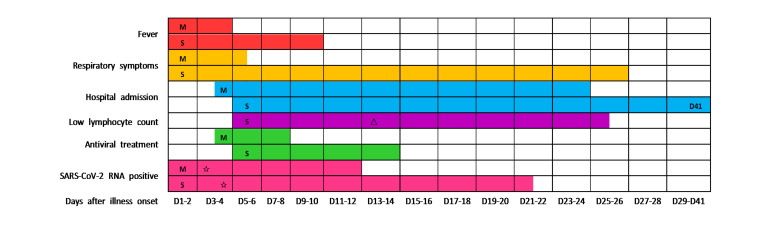
Major clinical courses in patients with COVID-19. Figure shows median duration of fever, respiratory symptoms, hospitalization, lymphopenia, antiviral treatment and viral carrying from illness onset of mild and severe patients. Most of the mild patients were not present lymphopenia, the median duration of lymphopenia in mild patients was 0, so the result was not shown in the figure. Low lymphocyte count (lymphopenia): lymphocyte count <0.8 × 10^9^/L. M: mild patients; S: severe patients; triangle: The lymphocyte count of severe patients was lowest on the day 13 after illness onset; star: The time point of confirmative examination of SARS-CoV-2; D41: The median duration of hospitalization for severe patients was 41 days.

The most common symptoms were fever, fatigue and cough, followed by polypnea, which was adherent to the majority of severe patients (**Table 1**). In addition, the duration of fever and respiratory symptoms was longer in severe group than that in mild (**Table 1** and **Figure 1**). Not only that, hypoxic symptoms had more priority in severe patients. A total of 36 (90.0%) patients had findings of bilateral infiltration on radiographic imaging (chest x-ray or computed tomography scan), while 2 (5.0%) patients had unilateral infiltration (**Table 1**).

### Laboratory findings

**Figure 2:**
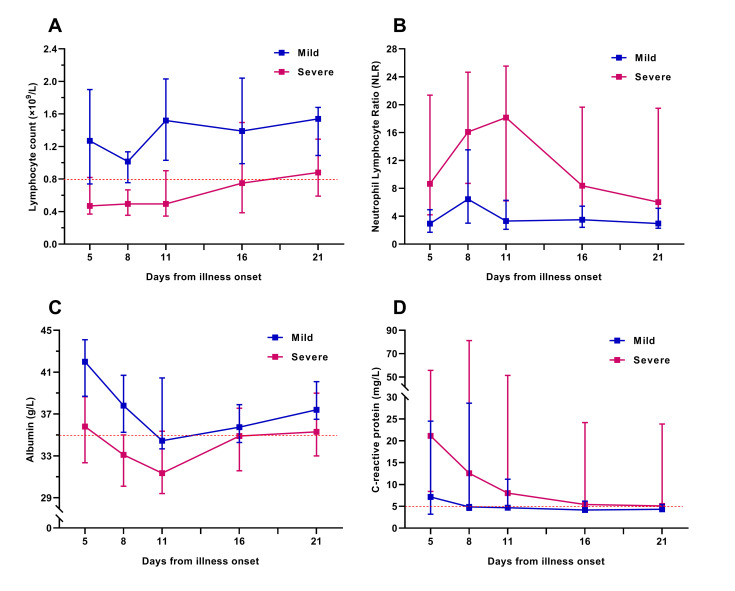
Dynamic changes in laboratory indicators during the hospitalization of patients with COVID-19. Figure shows the dynamic changes in lymphocyte count (**Panel A**), neutrophil lymphocyte ratio (**Panel B**), serum albumin (**Panel C**), and C-reactive protein (**Panel D**). Differences between the mild and the severe group were significant for all time points shown, except for serum albumin and C-reactive protein on the day 16 and day 21 after illness onset. Not all indicators of all patients were available at every time point.

We tracked the changes in laboratory indexes of all patients from hospital admission to discharge or death. Initial lymphocyte count on admission in severe group was significantly lower than that of the mild. Lymphopenia occurred in almost all severe patients during hospitalization, whereas in less than half of the mild (**Table 1**). In the severe patients, lymphocyte count was lowest on day 13 after illness onset and the lymphopenia could last for more than 20 days (**Figure 1**). Approximately one-third of all patients got a decrease of serum albumin on admission, which was more common in severe patients. The initial values of neutrophil count, neutrophil lymphocyte ratio (NLR), alanine aminotransferase, aspartate aminotransferase, and C-reactive protein (CRP) were higher in severe patients than in mild (**Table 1**). The dynamic changes of lymphocyte count, NLR, albumin, and CRP during hospitalization in mild and severe patients were elaborated by line chart (**Figure 2**).

Most severe patients underwent different degrees of anemia during hospitalization, but which was extremely rare in mild patients. Furthermore, LDH, fibrin degradation product (FDP), and D-dimer during the clinical course were significantly higher in severe patients than in mild, and which were far beyond the range of reference values. Given these laboratory tests were not performed within 48 hours after admission in some mild patients, there was no initial value for contrastive analysis between the two groups. (The differences of extreme values between the two groups were showed in Table S2 of the **Online Supplementary Document**)

### Treatments and clinical outcomes

Of all the enrolled patients, 2 (5.0%) severe patients died during hospitalization and the rest were recovered and discharged. The median duration of hospitalization for all discharged patients was 26.5 (16.5-37.5) days, which for severe and mild was 37.0 (26.0-42.0) days and 17.0 (12.0-27.0) days, respectively (**Table 1** and **Figure 1**). Approximately three-quarters of the enrolled patients received at least one mode of oxygen treatments, the most common of which was high-flow nasal cannula oxygen therapy. 13 (61.9%) severe patients received mechanical ventilation, while none of the mild. 39 (97.5%) patients received antiviral treatment (Lopinavir/Ritonavir or Arbidol), with a median duration of 7.5 (4.0-10.0) days. The median interval from illness onset to viral clearance in severe patients was 21.0 (13.0-41.0) days, which was longer than that of the mild (**Figure 1**). It must be noted, however, the virus was continuously detectable until death in 2 non-survivors. Of all the severe patients, the most common complications were ARDS and respiratory failure, followed by secondary infection. More detailed information about treatments and clinical outcomes were showed in **Table 1**.

### Early predictors and the prediction model for severe COVID-19

The univariate logistic regression analysis showed that the age, comorbidity, hypertension, lymphocyte count, neutrophil count, NLR, albumin and CRP were associated with the disease severity of COVID-19. Subsequently, all the above parameters with statistical significance in the univariate analysis were incorporated into the multivariate logistic regression model for in-depth analysis. Considering the relatively small sample size and the possibility of overfitting in the multivariate logistic regression model, we adopted a forward stepwise method (probability for stepwise: entry *P* < 0.05, removal *P* > 0.1) for logistic regression analysis to reduce the number of independent variables entering the model, so as to reduce the probability of model overfitting. The results showed that lymphocyte count and albumin on admission were the independent early predictors for severe COVID-19 (**Table 2**), and the severe COVID-19 risk model was constructed as following: Logit(*P*) = 15.779 − 2.531 × Initial lymphocyte count ( × 10^9^/L)  − 0.346 × Initial albumin (g/L).

**Table 2:** Univariate and multivariate logistic regression analysis* of risk factors associated with severe COVID-19

	Univariable OR (95% *CI*)	*P* value	Multivariable OR (95% *CI*)	*P* value
Age, years	1.063 (1.012-1.117)	0.014	1.010 (0.916-1.115)	0.837
Comorbidity present (yes vs no)	7.500 (1.798-31.283)	0.006	4.809 (0.577-40.052)	0.146
Hypertension (yes vs no)	4.848 (1.080-21.758)	0.039	1.400 (0.065-30.067)	0.830
Lymphocyte count, ×10^9^/L	0.040 (0.005-0.353)	0.004	0.080 (0.008-0.844)	0.036
Neutrophil count, ×10^9^/L	1.396 (1.044-1.868)	0.025	1.351 (0.873-2.089)	0.177
Neutrophil lymphocyte ratio	1.308 (1.059-1.615)	0.013	0.955 (0.558-1.633)	0.866
Albumin, g/L	0.663 (0.516-0.852)	0.001	0.707 (0.535-0.934)	0.015
Alanine aminotransferase, U/L	1.033 (0.998-1.069)	0.054	-	-
Aspartate aminotransferase, U/L	1.055 (0.999-1.098)	0.053	-	-
C-reactive protein, mg/L	1.043 (1.000-1.088)	0.048	1.005 (0.915-1.103)	0.925

*Severe COVID-19 risk model (constructed by multivariate logistic regression analysis): Logit(*P*) = 15.779 − 2.531 × Initial lymphocyte count ( × 10^9^/L)−0.346 × Initial albumin (g/L).

**Figure 3:**
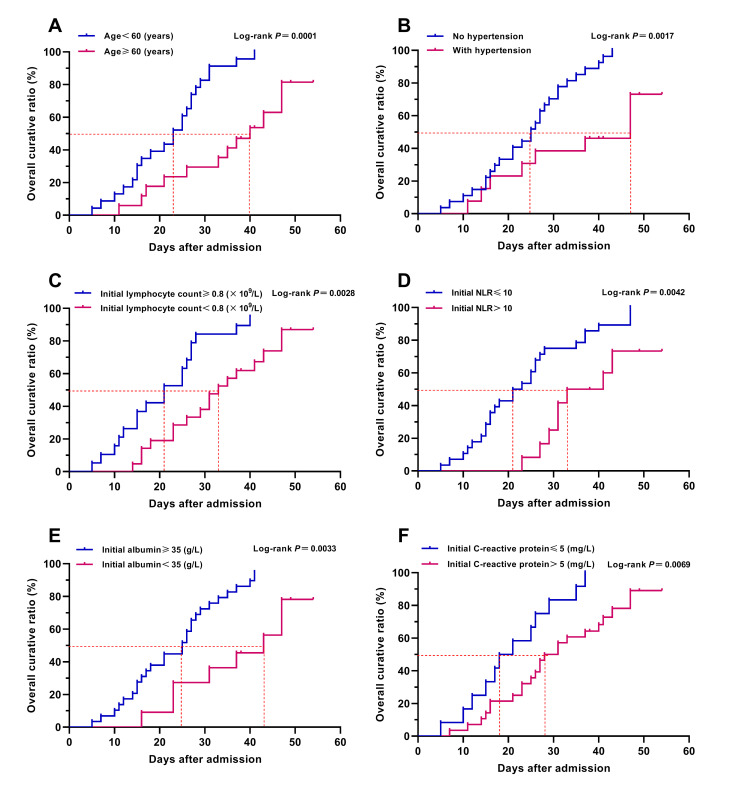
Kaplan-Meier survival analyses of major risk factors. Figure shows the Kaplan-Meier survival curves of age (**Panel A**), hypertension (**Panel B**), initial lymphocyte count (**Panel C**), initial NLR (**Pane D**), initial albumin (**Panel E**), and initial C-reactive protein (**Panel F**). Parameters of Kaplan-Meier survival analysis: events: discharge or death; time to events: time from hospital admission to discharge or death. Overall curative ratio was calculated by the number of discharged patients.

In order to reduce overfitting of the model, we adopted the forward stepwise method mentioned above for logistic regression analysis to reduce the number of independent variables entering the model, while some important predictors might be excluded from the small sample size logistic regression model and be identified as non-independent predictors. For the above reason, we also conducted Kaplan-Meier survival analysis and COX regression analysis to assess the effect of the above indicators on the prognosis of patient with COVID-19, so as to explore the potential independent predictors for severe COVID-19. The results of Kaplan-Meier survival curves with log-rank test showed that venerable age (≥60 years old), comorbidity, hypertension, lymphopenia, hypoalbuminemia, elevated NLR and CRP could obstruct the recovery and discharge of patients (**Table 3** and **Figure 3**). Further univariate and multivariate COX regression analysis showed that venerable age (≥60 years old), hypertension, lymphopenia, and elevated NLR were the independent adverse factors affecting the recovery and discharge of patients with COVID-19 (**Table 3**).

**Table 3:** Kaplan-Meier survival analysis and COX regression analysis of the adverse factors on the recovery and discharge of patient with COVID-19

	K-M survival analysis*	Univariate COX analysis*		Multivariate COX analysis*	
**log-rank test *P* value**	**HR (95% CI)**	***P* value**	**HR (95% CI)**	***P* value**
Age, years	-	0.949 (0.926-0.973)	<0.001	0.963 (0.937-0.989)	0.007
≥60 vs <60	<0.001	0.225 (0.099-0.511)	<0.001	-	-
Comorbidity present (yes vs no)	0.001	0.274 (0.125-0.602)	0.001	0.665 (0.189-2.345)	0.526
Hypertension (yes vs no)	0.002	0.257 (0.103-0.641)	0.004	0.312 (0.105-0.927)	0.036
Lymphocyte count, ×10^9^/L	-	3.145 (1.764-5.608)	<0.001	2.770 (1.256-6.110)	0.012
<0.8 vs ≥0.8	0.003	0.349 (0.169-0.724)	0.005	-	-
Neutrophil count, ×10^9^/L	-	0.837 (0.733-0.955)	0.008	1.112 (0.806-1.536)	0.518
>7.5 vs 2.5-7.5	0.139	0.472 (0.166-1.339)	0.158	-	-
Neutrophil lymphocyte ratio	-	0.906 (0.853-0.963)	0.001	0.937 (0.870-1.010)	0.091
>10.0 vs ≤10.0	0.004	0.335 (0.151-0.747)	0.007	-	-
Albumin, g/L	-	1.157 (1.078-1.242)	<0.001	1.046 (0.940-1.164)	0.409
<35.0 vs ≥35.0	0.003	0.261 (0.099-0.689)	0.007	-	-
C-reactive protein, mg/L	-	0.973 (0.954-0.993)	0.008	0.993 (0.973-1.014)	0.512
>5 vs ≤5	0.007	0.377 (0.179-0.793)	0.010	-	-

K-M survival analysis – Kaplan-Meier survival analysis, COX analysis – COX regression analysis

*Events: discharge or death; Time to Events: time from hospital admission to discharge or death.

Through the combination utilization of the statistical analysis methods mentioned above, we finally ascertained that venerable age (≥60 years old), hypertension, lymphopenia, hypoalbuminemia and elevated NLR were the independent high-risk factors associated with the disease severity of COVID-19, and the age, initial lymphocyte count, initial albumin and initial NLR could severe as the independent early predictors for severe COVID-19. In addition, the severe COVID-19 risk model (constructed by multivariate logistic regression analysis) might be a helpful tool for the early prediction and identification of severe patients during the pandemic of COVID-19.

### Predictive efficacy of the severe COVID-19 risk model and early predictors

**Figure 4:**
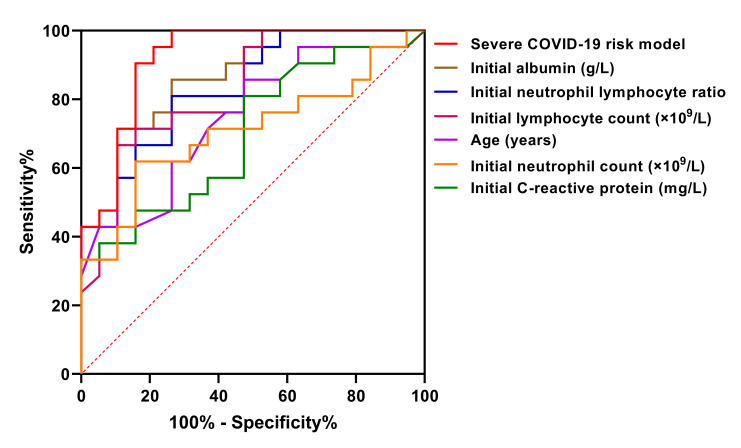
ROC curves of severe COVID-19 risk model and early predictors. Figure shows the predictive efficacy of the severe COVID-19 risk model and early predictors by ROC curves. Severe COVID-19 risk model: constructed by multivariate logistic regression analysis.

ROC curves were used to assess the predictive efficacy of the severe COVID-19 risk model and each early predictor. According to the order of area under ROC curve from large to small, these early predictors were the severe COVID-19 risk model (0.920), albumin (0.867), NLR (0.835), lymphocyte count (0.826), and age (0.747), successively (**Table 4** and **Figure 4**). The predictive value of the severe COVID-19 risk model which combined with multiple parameters was the best, whose sensitivity and specificity was 90.5% and 84.2%, respectively, and its positive predictive value, negative predictive value and crude agreement were all over 85% (**Table 4**).

**Table 4:** Predictive efficacy* of the severe COVID-19 risk model and early predictors

	AUC (95% CI)	*P* value	Sensitivity	Specifity	Cut-off value	Crude agreement	Youden’s index	Positive predictive value	Negative predictive value	Positive likelihood ratio	Negative likelihood ratio
Severe COVID-19 risk model†	0.920 (0.832-1.000)	<0.001	90.5%	84.2%	-0.232	87.5%	0.747	86.4%	88.9%	5.728	0.113
Albumin, g/L	0.867 (0.759-0.976)	<0.001	89.5%	71.4%	37.75	80.0%	0.609	88.2%	73.9%	3.129	0.147
Neutrophil lymphocyte ratio	0.835 (0.713-0.956)	<0.001	81.0%	73.7%	3.84	77.5%	0.547	77.3%	77.8%	3.080	0.258
Lymphocyte count, ×10^9^/L	0.826 (0.697-0.954)	<0.001	89.5%	66.7%	0.62	77.5%	0.562	87.5%	70.8%	2.688	0.157
Age, years	0.747 (0.595-0.899)	0.008	85.7%	52.6%	44.5	70.0%	0.383	66.7%	76.9%	1.808	0.272
Neutrophil count, ×10^9^/L	0.709 (0.546-0.873)	0.024	61.9%	84.2%	4.525	72.5%	0.461	81.3%	66.7%	3.918	0.452
C-reactive protein, mg/L	0.699 (0.537-0.862)	0.031	81.0%	52.6%	7.715	67.5%	0.336	65.4%	71.4%	1.709	0.361

AUC – area under ROC curve

*The predictive efficacy was evaluated by receiver operating characteristic (ROC) curves.

†Severe COVID-19 risk model: constructed by multivariate logistic regression analysis.

## DISCUSSION

The exponential increasing number of patients with COVID-19 has brought a heavy burden to the medical health service systems in countries with large outbreaks. As reported by Zhang et al [17], the effective triage and hierarchical medical system and timely supplement of medical resources played an important role on reducing the mortality of COVID-19 in the pandemic inundated regions. Therefore, it is essential to ascertain the early predictors which could help clinicians to identify the severity of patients with COVID-19 quickly at an early stage. In this retrospective cohort study, we identified several independent risk factors for severe COVID-19, which were venerable age, hypertension, lymphopenia, hypoalbuminemia and elevated NLR. Some of these risk factors have been identified in previous studies, while the hypoalbuminemia and elevated NLR have not been reported so far.

The majority of previous studies have reported that elderly patients with COVID-19 were more likely to progress to severe stage and the mortality of elderly patients was higher than that of the young and middle-aged [3-14]. The results of our study also confirmed that venerable age was an independent risk factor of severe COVID-19. The weakness of immunity and the decline of compensatory function of visceral organs in elderly patients may be one of the reasons why they were prone to develop severe COVID-19 after SARS-CoV-2 infection.

Lymphopenia is a widespread manifestation in patients with COVID-19, especially in severe patients and the deceased. In a multicenter study which described the clinical characteristics of 1099 patients with COVID-19, lymphopenia was reported in 82.1% of patients [4]. In present study, we not only reported that lymphopenia was associated with the severity of COVID-19, but also compared the dynamic changes of lymphocyte count during hospitalization in mild and severe patients. The lower lymphocyte count and the longer duration of lymphopenia, the condition of patients with COVID-19 may more serious and the prognosis of whom might more worse.

Similarly, the elevation of neutrophils in patients with COVID-19 has been reported in several previous studies. Wu et al [12] have reported that neutrophilia is a risk factor associated with the development of ARDS and progression from ARDS to death in patients with COVID-19. In our study, although the neutrophil count might be associated with the severity of COVID-19 in univariate analysis, multivariate analysis indicated that it was not an independent early predictor for severe COVID-19, and the results of ROC curve analysis also showed that the predictive efficacy of which was unsatisfactory. In view of the relatively limited predictive efficacy of the lymphocyte count and neutrophil count, we innovatively introduced neutrophil lymphocyte ratio (NLR) to statistical analysis. Excitingly, the results showed that NLR was not only an independent early predictor for severe COVID-19, which also got a better predictive performance than lymphocyte count.

In addition, several previous studies have shown that patients with COVID-19 were often accompanied by albumin reduction, and the level of serum albumin in severe patients were lower than that in mild [3,12-14]. Coincidentally, all of these studies did not elucidate the role of albumin in the prediction on disease severity or prognosis of COVID-19. Considering that the therapeutic measures such as intravenous albumin infusion may mask the true serum albumin levels of patients with COVID-19, we selected the initial albumin on admission for analysis. The results showed that the initial albumin on admission was an independent early predictor with a good predictive performance for severe COVID-19. Several previous studies have showed that not only capable of causing pneumonia, COVID-19 may also cause damage to other organs such as the heart, the liver, and the kidneys, as well as to organ systems such as the blood and the immune system [3,13-16]. Based upon our results and the clinical experience about COVID-19, we conjectured that the decrease of albumin may be related to the low nutritional status, hypo-function of liver synthesis and acute kidney injury after SARS-CoV-2 infection. For all that, the mechanism of serum albumin reduction is still unclear and need further study.

On the aspect of the viral dynamic changes, Liu et al [18] reported that the mean viral load of severe cases was around 60 times higher than that of mild, which indicated that higher viral loads might be associated with severe clinical outcomes. In present study, we elaborated the details of virological transformation course in patients with COVID-19. We observed that the duration of viral carrying (from illness onset to viral clearance) was longer in severe patients than that in mild, and the virus was continuously detectable until death in 2 non-survivors. The above findings of us coincided with the results of Zhou et al [14], and the later described the duration of viral shedding in survivals and non-survivals for the first time. The early viral clearance strategy might be benefit to the recovery of patients with COVID-19 and reduce the transmission probability of SARS-CoV-2 [19]. All the above findings on virological transformation course might have important implications on patient isolation decision making and the guidance around the length of antiviral treatment.

This study has several limitations. First, this is a single-center, small-sample, retrospective study. There were a total of 120 COVID-19 patients in Xi'an City, and only 40 of them were treated in our center which was one of the designated hospitals for COVID-19 patients in Xi'an. Considering this was a retrospective study and the relatively small number of patients in our center, so we enrolled all patients into the analysis data set without calculating the sample size in advance. Given the small sample size of this study, the continuous variables between the mild group and the severe group were compared by Mann-Whitney U test, which may reduce the statistical power of the analysis. Due to the small sample size, our logistic model only incorporated two early predictors and excluded some important predictors such as age. For the above reasons, the findings of us might be limited by the sample size. Second, not all laboratory indicators were detected in all patients, and not all laboratory tests should be performed immediately after admission. Therefore, the initial values of some laboratory indicators such as LDH, IL-6, procalcitonin, FDP and D-dimer, were absent in several mild patients, and the predictive value of which were not evaluated in this study. Third, there was a great possibility of overfitting in the severe COVID-19 risk model which we constructed, so the prospective cohort studies are needed to further confirm the reliability of the early predictors and to construct a new predictive model or scoring criteria of severe COVID-19 for clinical application in the future. Last but not least, the estimated duration of viral carrying might be limited by the frequency of respiratory specimen collection and the lack of quantitative viral RNA detection.

## CONCLUSIONS

In summary, we elaborated the clinical features and virological transformation course of COVID-19, identified several independent early predictors (age, lymphocyte count, albumin, NLR), and constructed a prediction model with a favorable predictive efficacy for severe COVID-19. All these findings may have important implications on the early warning of severe COVID-19, the decision making of patient isolation, and the guidance around the length of antiviral treatment, and which may help clinicians to identify the severe patients quickly at an early stage and reasonably allocate medical resources, so as to improve the therapeutic effect of severe COVID-19.

## Additional material

Online Supplementary Document
