# Supplementary material for: Early prediction and identification for severe patients during the pandemic of COVID-19: A severe COVID-19 risk model constructed by multivariate logistic regression analysis
Source: J Glob Health. 2020 Sep 4;10(2):020510. doi: 10.7189/jogh.10.020510 (PMC7567445; doi:10.7189/jogh.10.020510)
Supplement: Online Supplementary Document [file jogh-10-020510-s001.pdf]

Online Supplementary Document

Table S1. Grouping criteria based on the clinical typing standard of COVID-19

Group	Clinical type	Clinical typing standard of COVID-19*
Mild group†	Mild type	Patients with slight clinical symptoms and no sign of pneumonia on imaging.
	Moderate type	Patients with fever, respiratory tract and other symptoms, and pneumonia changes on imaging.
Severe group‡	Serious type	Patients presenting with any of the following: (1) polypnea, respiratory rate ≥30/min; (2) finger oxygen saturation ≤93% in resting condition; (3) arterial partial pressure of oxygen (PaO_2)/fraction of inspired oxygen (FiO_2) ≤300mmHg (1mmHg=0.133kPa); (4) pulmonary imaging demonstrates that the lesions expanded rapidly more than 50% in 24~48 hours.
	Critical type	Patients meeting any of the following: (1) respiratory failure and requiring for mechanical ventilation; (2) shock; (3) accompanying with other organ failure and needing for ICU care and treatment.

*The clinical typing standard of COVID-19 were derived from the "Diagnosis and Treatment Protocol for Novel Coronavirus Pneumonia (Trial Version 7)" issued by the National Health Commission of China.

†The mild group included all patients of mild type and moderate type.

‡The severe group included all patients of serious type and critical type.

Table S2. Differences of extreme values* of laboratory parameters between the two groups

	All patients (*n*=40)	Mild group (*n*=19)	Severe group (*n*=21)	*P* value
Maximum white blood cell count, $\times 10^9$/L	10.99 (7.94−16.18)	8.26 (7.01−12.74)	14.07 (9.85−18.83)	0.006
>10	22 (55.0%)	6 (31.6%)	16 (76.2%)	0.005
Minimum lymphocyte count, $\times 10^9$/L	0.55 (0.31−1.07)	0.83 (0.57−1.58)	0.32 (0.26−0.52)	<0.001
<0.8	27 (67.5%)	8 (42.1%)	19 (90.5%)	0.001
Maximum neutrophil count, $\times 10^9$/L	8.77 (6.39−14.68)	6.51 (4.96−10.13)	12.04 (8.18−17.35)	0.002
>7.5	24 (60.0%)	7 (36.8%)	17 (81.0%)	0.004
Maximum neutrophil lymphocyte ratio	13.51 (4.50−25.15)	7.32 (3.52−12.27)	23.91 (14.58−60.65)	<0.001
Minimum platelet count, $\times 10^9$/L	147.50 (105.00－183.75)	185.00 (149.00－226.50)	104.00 (70.00－153.00)	0.001
<100	9 (22.5%)	0 (0.0%)	9 (42.9%)	0.001
Minimum haemoglobin, g/L	123.00 (106.75－134.50)	128.00 (124.00－137.00)	98.00 (79.00－123.00)	<0.001
<120	17 (42.5%)	2 (10.5%)	15 (71.4%)	<0.001
Minimum albumin, g/L	32.30 (29.13−35.80)	35.80 (33.60−40.30)	29.20 (27.60−31.75)	<0.001
<35	28 (70.0%)	9 (47.4%)	19 (90.5%)	0.003
Maximum globulin, g/L	34.95 (32.03−39.45)	33.30 (31.30−36.00)	38.30 (33.80−44.85)	0.007
>32	30 (75.0%)	12 (63.2%)	18 (85.7%)	0.148
Maximum alanine aminotransferase, U/L	95.00 (45.25−143.50)	50.00 (37.00−98.00)	124.00 (93.00−195.00)	0.001
>40	31 (77.5%)	11 (57.9%)	20 (95.2%)	0.007
Maximum aspartate aminotransferase, U/L	56.00 (34.25−81.75)	35.00 (33.00−52.00)	72.00 (57.00−120.50)	<0.001
>40	25 (62.5%)	7 (36.8%)	18 (85.7%)	0.001
Maximum total bilirubin, μmol/L	22.79 (14.13−39.40)	17.10 (13.00−22.38)	34.80 (20.70−57.15)	0.001
>17.1	24 (60.0%)	8 (42.1%)	16 (76.2%)	0.028
Maximum blood urea nitrogen, mmol/L	6.45 (4.97−9.64)	5.87 (4.40−7.07)	7.44 (5.90−18.34)	0.006
>7.5	12 (30.0%)	2 (10.5%)	10 (47.6%)	0.011
Maximum creatinine, μmol/L	77.95 (62.33−89.98)	77.20 (62.70−80.60)	85.90 (57.80−97.10)	0.189
>104	5 (12.5%)	1 (5.3%)	4 (19.0%)	0.345
Maximum C-reactive protein, mg/L	25.07 (8.38−59.17)	20.30 (4.20−35.55)	54.56 (9.35−105.55)	0.031
>5	33 (82.5%)	14 (73.7%)	19 (90.5%)	0.226
†Maximum creatine kinase, U/L	59.00 (38.00−106.00)	45.00 (37.00−85.00)	67.00 (38.00−187.25)	0.393
>198	4 (11.4%)	0 (0.0%)	4 (19.0%)	0.133
†Maximum creatine kinase MB, U/L	12.50 (8.00−15.95)	8.50 (8.00−13.20)	13.80 (8.50−19.70)	0.041
>18	5 (14.3%)	0 (0.0%)	5 (23.8%)	0.069
†Maximum lactate dehydrogenase, U/L	562.0 (400.5−794.0)	414.0 (308.0−544.0)	715.0 (540.25−1047.75)	0.002
>245	27 (77.1%)	9 (64.3%)	18 (85.7%)	0.221
†Maximum prothrombin time, s	11.90 (11.40−14.35)	11.35 (11.20−12.55)	12.50 (11.80−16.30)	0.118
>13.0	9 (26.5%)	1 (7.7%)	8 (38.1%)	0.107
†Maximum fibrinogen, g/L	4.82 (4.55−5.69)	4.55 (3.85−5.43)	5.06 (4.71−5.76)	0.035

>4.0	26 (76.5%)	7 (53.8%)	19 (90.5%)	0.033
†Maximum fibrin degradation product, mg/L	6.70 (4.00−23.85)	3.80 (2.85−4.25)	19.50 (6.40−62.30)	<0.001
>5.0	17 (50.0%)	1 (7.7%)	16 (76.2%)	<0.001
†Maximum D-dimer, mg/L	2.78 (1.22−11.01)	1.14 (0.94−1.46)	8.12 (2.78−21.74)	<0.001
>0.5	29 (85.3%)	10 (76.9%)	19 (90.5%)	0.348
‡Albuminuria	19 (47.5%)	4 (21.1%)	15 (71.4%)	0.001
‡Hematuria	13 (32.5%)	2 (10.5%)	11 (52.4%)	0.005
‡Urine leukocyte	5 (12.5%)	1 (5.3%)	4 (19.0%)	0.345

Continuous variables were presented as median (interquartile range) and compared by Mann-Whitney U test.

Categorical variables were presented as numbers (percentage) and compared by Fisher's exact test.

*Extreme value referred to the maximum or minimum value of laboratory tests during hospitalization.

†These laboratory indicators were detected in only 34 cases.

‡Albuminuria, hematuria, and urine leukocyte were from routine urinalysis during hospitalization.
